
JOURNAL INFORMATION
==============================
NLM Title Abbreviation: ScientificWorldJournal
Journal ID: ScientificWorldJournal
NLM Journal ID: 101131163
Journal ID: TSWJ

The Scientific World Journal

ISSN: 2356-6140
EISSN: 1537-744X
Publisher: Wiley

ARTICLE INFORMATION
==============================
PMCID: PMC6084144
DOI: 10.1100/tsw.2001.432
Article ID: 419862
Article version: 1
Subjects: Extended Abstract

Distribution of Receptor Activity Modifying Protein (RAMP) mRNAS in the Rat Central Nervous System

Oliver K. R.
Kinsey A. M.
Keyvan-Fouladi N.
Heavens R.
Wainwright A.
Merck Research Laboratories, Neuroscience Research Centre, Harlow, Essex CM20 2QR, UK
Publication date: 2001
Electronic publication date: 2001 Jun 16
Volume: 1
First page: 45
Last page: 45
Copyright: Copyright © 2001 K. R. Oliver et al.
Copyright year: 2001
License: This is an open access article distributed under the Creative Commons Attribution License, which permits unrestricted use, distribution, and reproduction in any medium, provided the original work is properly cited.
License URL: https://creativecommons.org/licenses/by/3.0/

==============================
4th International Meeting on Calcitonin Gene-Related Peptide (CGRP)
*TheScientificWorld* (2001) 1(S1), 45
ISSN 1532-2246; DOI 10.1100/tsw.2001.432

### DISTRIBUTION OF RECEPTOR ACTIVITY MODIFYING PROTEIN (RAMP) mRNAS IN THE RAT CENTRAL NERVOUS SYSTEM

K.R. Oliver, A.M. Kinsey, N. Keyvan-Fouladi, R. Heavens, and A. Wainwright
Merck Research Laboratories, Neuroscience Research Centre, Harlow, Essex CM20 2QR, U.K.

Calcitonin gene-related peptide (CGRP), adrenomedullin, amylin, and calcitonin are structurally and functionally related neuropeptides. It is known that the molecular pharmacology of CGRP and adrenomedullin is determined by coexpression of one of three receptor activity-modifying proteins (RAMPs) with calcitonin receptor-like receptor (CRLR). Furthermore, RAMPs also govern the pharmacology of the calcitonin receptor, which in association with RAMP1 or RAMP3, binds amylin with high affinity. Through sequencing and cloning of the rat RAMP isologues, and using specific oligonucleotides to each, we have determined the expression of RAMP1, RAMP2, and RAMP3 mRNAs in the rat central nervous system by *in situ* hybridization. The localization of RAMP mRNAs was heterogeneous. RAMP1 mRNA was predominantly expressed in cortex, caudate putamen, and olfactory tubercles; RAMP2 mRNA was most abundant in hypothalamus; and RAMP3 mRNA was restrictively expressed in thalamic nuclei. Interestingly, in specific brain areas only a single RAMP mRNA was often detected, suggesting mutual exclusivity in expression. These data allow predictions to be made of where each RAMP protein may heterodimerize with its partner G-protein coupled receptor(s) at the cellular level and consequently advance current understanding of cellular sites of action of CGRP, ADM, amylin, and calcitonin. Furthermore, these localization data suggest that the RAMP family may associate and modify the activity of other, as yet unidentified neurotransmitter receptors.
